# Maize-soybean relay strip intercropping reshapes the rhizosphere bacterial community and recruits beneficial bacteria to suppress *Fusarium* root rot of soybean

**DOI:** 10.3389/fmicb.2022.1009689

**Published:** 2022-10-26

**Authors:** Xiaoli Chang, Dengqin Wei, Yuhan Zeng, Xinyu Zhao, Yu Hu, Xiaoling Wu, Chun Song, Guoshu Gong, Huabao Chen, Chunping Yang, Min Zhang, Taiguo Liu, Wanquan Chen, Wenyu Yang

**Affiliations:** ^1^College of Agronomy and Sichuan Engineering Research Center for Crop Strip Intercropping System, Sichuan Agricultural University, Chengdu, China; ^2^State Key Laboratory for Biology of Plant Diseases and Insect Pests, Institute of Plant Protection, Chinese Academy of Agricultural Sciences, Beijing, China

**Keywords:** maize-soybean relay strip intercropping, soybean root rot, rhizosphere microbial community, plant growth promotion bacteria, *Pseudomonas chlororaphis*

## Abstract

Rhizosphere microbes play a vital role in plant health and defense against soil-borne diseases. Previous studies showed that maize-soybean relay strip intercropping altered the diversity and composition of pathogenic *Fusarium* species and biocontrol fungal communities in the soybean rhizosphere, and significantly suppressed soybean root rot. However, whether the rhizosphere bacterial community participates in the regulation of this intercropping on soybean root rot is not clear. In this study, the rhizosphere soil of soybean healthy plants was collected in the continuous cropping of maize-soybean relay strip intercropping and soybean monoculture in the fields, and the integrated methods of microbial profiling, dual culture assays *in vitro*, and pot experiments were employed to systematically investigate the diversity, composition, and function of rhizosphere bacteria related to soybean root rot in two cropping patterns. We found that intercropping reshaped the rhizosphere bacterial community and increased microbial community diversity, and meanwhile, it also recruited much richer and more diverse species of *Pseudomonas* sp., *Bacillus* sp., *Streptomyces* sp., and *Microbacterium* sp. in soybean rhizosphere when compared with monoculture. From the intercropping, nine species of rhizosphere bacteria displayed good antagonism against the pathogen *Fusarium oxysporum* B3S1 of soybean root rot, and among them, IRHB3 (*Pseudomonas chlororaphis*), IRHB6 (*Streptomyces*), and IRHB9 (*Bacillus*) were the dominant bacteria and extraordinarily rich. In contrast, MRHB108 (*Streptomyces virginiae*) and MRHB205 (*Bacillus subtilis*) were the only antagonistic bacteria from monoculture, which were relatively poor in abundance. Interestingly, introducing IRHB3 into the cultured substrates not only significantly promoted the growth and development of soybean roots but also improved the survival rate of seedlings that suffered from *F. oxysporum* infection. Thus, this study proves that maize-soybean relay strip intercropping could help the host resist soil-borne *Fusarium* root rot by reshaping the rhizosphere bacterial community and driving more beneficial microorganisms to accumulate in the soybean rhizosphere.

## Introduction

Faced with the explosive growth of the world population, it is estimated that the need for agriculture production is increased by 70% by 2050 (Jez et al., 2016). One of the biggest agricultural challenges in the twenty-first century is that the production of stable crops is very hard to sufficiently satisfy the globally growing demand for food (Tilman et al., 2002). The rational use of legumes and grains cultivation is among the important agricultural measures in line with the sustainable development strategy and has big advantages on the full and effective use of limited land resources and the increase of grain yield (Pradhan et al., 2015). Recently, maize-soybean relay strip intercropping has been creatively developed in Southwest China, which is typically composed of two-row maize plants intercropped with two-to-four rows of soybean rather than traditional maize-soybean intercropping (Du et al., 2018). A series of studies have shown that maize-soybean relay strip intercropping plays a vital role in the effective use of locally limited solar-thermal resources, increasing land equivalent ratio (Pradhan et al., 2015; Yang et al., 2015), improving soil quality (Tanveer et al., 2017), promoting yield stability (Du et al., 2018), and minimizing damage caused by diseases (Gao et al., 2014) and weeds (Su et al., 2018). At present, this cropping pattern has received widespread attention and is practiced widely in China and other Asian countries. Particularly, continuous cropping of maize-soybean relay strip intercropping largely has reshaped the pathogen population and weaken their pathogenicity, thus contributing to a decline of soybean root rot in the fields (Chang et al., 2018, 2020). However, the regulation mechanism of this cropping on soybean root rot is largely not elucidated.

The rhizosphere is the core area where soil-borne diseases occur, and it is also the hot spot of the interaction among microorganisms, pathogens, and plants (Raaijmakers et al., 2008). It is generally believed that the rhizosphere microbial community is closely related to plant health, and it can also be used as an ecological barrier to limit pathogen invasion (Hacquard et al., 2017). In the rhizosphere, 95% of microorganisms belong to bacteria (Timmusk et al., 2017). Rhizosphere bacteria are often capable of colonizing the ecological niches on the roots at all stages of plant growth (Antoun and Prévost, 2005). Plant growth promoting rhizosphere bacteria (PGPR) is one kind of important beneficial rhizosphere microorganism for plant health (Timmusk et al., 2017), and they generally account for 2–5% of rhizobacteria (Kumar et al., 2012). PGPR can promote plant growth by producing indole-3-acetic acid (IAA), nitrogen fixation, and dissolving soil phosphorus and various nutrients to directly serve hosts, and on the other hand, they can also inhibit or kill pathogens by producing iron carriers, cellulose, protease, antibiotics, and cyanide to indirectly protect plants (Glick, 1995). Many scientists have reported that the PGPR belongs to *Bacillus* sp., *Pseudomonas* sp., and *Streptomyces* sp., and some of these microorganisms have been explored as biocontrol agents to help crops, such as maize (Song et al., 2021), pepper (Hyder et al., 2020), tomato (Beris et al., 2018), soybean (Kumar et al., 2012), and banana (Tao et al., 2020), resist soil-borne diseases. Moreover, of the complex interactions within the microbial community, high diversity tends to increase the community's resistance to pathogen invasion (Wei et al., 2015). Previous studies have demonstrated that intercropping can regulate the rhizosphere microbial community, improve microbial diversity, and restrict the invasion and development of pathogens, thus effectively reducing the occurrence of soil-borne diseases (Ratnadass et al., 2012; Yang et al., 2014). Especially, long-term continuous cropping often decreases the rhizosphere microbial diversity (Tan et al., 2017), accumulates pathogen inoculum, and affects the plant health, thus leading to the reduction of crop yield (Zhao et al., 2021). However, the current data on how the maize-soybean relay strip intercropping affects the rhizosphere microbial community and especially how the abundance and diversity of beneficial rhizosphere bacteria are regulated to help soybean resist soil-borne diseases are not uncovered.

In this study, we collected the rhizosphere soil of healthy soybean plants under maize-soybean relay strip intercropping and soybean monoculture and carried out a series of experiments, including high-throughput sequencing, traditional microbial isolation, screening of antagonistic bacteria, and pot experiments. By comparing the composition and function of rhizosphere microorganisms in two cropping patterns, we make it clear (1) whether the maize-soybean intercropping changes the composition and diversity of the rhizobacteria community; (2) how this intercropping affects the beneficial bacteria genera of rhizosphere soil; and (3) how these beneficial bacteria help plants resist soil-borne diseases. We hope this study will provide some references for a better understanding of rhizosphere microbial regulation on soil-borne disease under maize-soybean relay strip intercropping which is helpful to formulate sustainable and environmentally friendly agricultural management strategies.

## Materials and methods

### Field experiments and collection of rhizosphere soil

Location field experiments of maize-soybean relay strip intercropping and soybean monoculture were planted in the Ya'an Experimental Farm of Sichuan Agricultural University (29°98N, 102°98E), Sichuan Province, China, according to Chang et al. (2020). The randomized complete block design was used with three replicated experimental plots for two cropping patterns. Each plot was designed for 6 m in width and 6 m in length. For soybean monoculture, 5-row soybeans were cultivated with a row distance of 0.5 m and a hole space of 0.34 m. For maize-soybean relay strip intercropping, one strip production unit contained two-row maize and two-row soybean in 2.0 m in width, and the row space distance was 0.4 m for maize rows or soybean rows and 0.6 m between maize and soybean rows. Each plot for intercropping covered three strip production units, and the hole space was 17 cm for both maize and soybean, respectively. Maize cultivar “Denghai605” was sown in late May and harvested in Mid-August, while soybean cultivar “Nandou12” was sown in Mid-June and harvested in late October. No tilling was performed before sowing every year.

Rhizosphere soil samples of healthy soybean plants were collected from both cropping patterns at the full bloom (R2) stage of soybean in August 2021 when soybean root rot frequently occurred in the fields, and the data of disease incidence and soybean growth are seen in Supplementary Figure S1. The rhizosphere soils of healthy soybean plants were obtained by gently shaking big soil blocks and collecting the soil tightly attached to the root surface about 1–5 mm. Each soil sample was derived from 40 soybean plants. All soil samples were taken to the laboratory with dry ice. One part of the samples was stored at −20°C for microbial isolation within 48 h, while other parts were stored at −80°C to perform high-throughput sequencing.

### Microbiota genome DNA extraction and sequencing

Genome DNA of rhizosphere soils was extracted by the SDS method, and the quality of DNA was monitored on 1% agarose gel. DNA was diluted to 1.0 ng·μl^−1^ with ddH_2_O according to their concentration. Fragments of the *16S rDNA* gene were amplified using the primer pairs 515F (5′-GTGYCAGCMGCCGCGGTAA-3′) and 806R (5′-GGACTACNVGGGTWTCTAAT-3′) (Tedersoo et al., 2020). PCR products were mixed with an equal volume of 1× sample buffer (including SYB green) for electrophoretic detection. The samples with 400–450 bp bright bands were selected for further experiments. PCR products were further mixed in equal density proportion and then purified with Qiagen Gel Extraction Kit (Qiagen, Germany). TruSeq^®^ DNA PCR-Free Sample Preparation Kit (Illumina, USA) was employed to generate the sequencing library. The quality evaluation of the sequencing library was conducted on Qubit@ 2.0 fluorometer (Thermo Scientific, USA) and Agilent Bioanalyzer 2100 systems. Finally, the library was sequenced on the Illumina HiSeq 2500 platform to generate 250 bp double terminal readings.

### Sequence processing

Based on the unique barcode, paired-end reads were assigned to samples. These reads were further modified by cutting off the barcode and primer sequence and merged using FLASH (V1.2.7, http://ccb.jhu.edu/software/FLASH/). Quality filtering of the raw tags was performed to obtain the high-quality clean tags through the QIIME quality-controlled process. After comparison with the reference database (Unite Database, https://unite.ut.ee/) using the UCHIME algorithm (http://www.drive5.com/usearch/manual/uchime_algo.html), chimera sequences were detected and then removed. The sequences with effective tags similarity ≥97% were clustered into operational taxonomic units using Uparse v7.0.1001 (http://drive5.com/uparse). Each representative sequence was annotated to taxonomic information which was calculated by QIIME software (Version 1.7.0) (http://qiime.org/scripts/assign_taxonomy.html), according to the Blast algorithm of Unite Database (https://unite.ut.ee/). The abundance information of OTUs was normalized using a standard sequence number corresponding to the sample with the least sequences. Subsequently, both alpha diversity and beta diversity were analyzed using this output normalized data. Alpha diversity was used to analyze the complexity of species diversity for a sample through four indices, including observed species, Chao 1, PD whole tree, and Shannon. All these indices were calculated with QIIME (Version 1.7.0) and displayed with R software 2.15.3. Beta diversity analysis on unweighted unifrac was calculated by QIIME to evaluate sample differences in species complexity. The significance of differences between sample clusters was assessed with Analysis of Similarity (ANOSIM).

### Isolation and identification of rhizosphere soil bacteria

In brief, a total of 10 g of soil was suspended in 90 ml of sterilized water and was shaken for 30 min at 150 rpm·min^−1^ to make a mixed suspension. After that, the suspension was placed for 30 s, and supernatants were collected and serially diluted to obtain dilutions from 10^−1^ to 10^−5^. About 100 μl of diluent was spread on Nutrient agar medium (NA, containing beef extract 3 g, tryptone 5 g, yeast extract 1 g, glucosum anhydricum 10 g, agar 15 g, sterilized water 1,000 ml, and pH 7.0) and then incubated at 28°C in an incubator (SHP-300, Sanfa Scientific Instruments, Shanghai) for 2–5 days. Colonies of bacteria were initially numbered and counted according to morphology, color, and margin. Representative types of bacterial colonies were picked from the dilute plate with the most abundant colonies and recultured on the new NA plates to obtain pure colonies. Purified bacterial strains were kept in 25% glycerol at −80°C.

For bacterial identification, colony PCR was performed to amplify *16S rDNA* gene fragments using primer pair as follows: 27F (5′-AGAGTTTGATCMTGGCTCAG-3′) and 1426R (5′-GGTTACCTTGTTACGACTT-3′) (Gupta et al., 1994). PCR reaction contained template DNA 1.0 μl, each primer 1.0 μl (10 μM), Taq PCR Mastermix (Sangon Biotech, Shanghai, China) 12.5 μl, and DNase free water 9.5 μl, which in total 25-μl volume. PCR amplification was conducted using the parameters as follows: initial denaturation at 95°C for 5 min, 34 cycles of 95°C for 1 min, 55°C for 30 s, and 72°C for 1 min, with a final extension at 72°C for 10 min. The PCR products were analyzed on 1.2% agarose gel electrophoresis, and the purified products were sequenced (Sangon Biotech, Shanghai, China). The sequences were analyzed using Blast analysis of the NCBI database (https://www.ncbi.nlm.nih.gov/) and the Ribosomal Database Project reference database (version 19) for bacterial taxa classification. All sequences have been submitted to the NCBI database (https://www.ncbi.nlm.nih.gov/nuccore/?term=OP364493:OP364720[accn]).

### Quantify cultural bacteria

Based on the colony numbers in different dilution above, the most abundant colonies in the corresponding dilution was used for bacterial quantification. The abundance of cultural bacteria was quantified according to soil water content (%) and colony units (cfu·g^−1^) by the formulas below.


$$\begin{array}{l} \text{ Soil water content }(\%)=\frac{\text{Fresh soil weight }-\text{ Fried soil weight}}{\text{Fried soil weight}}\times 100\\ \text{Colony units }(\text{cfu·}{\text{g}}^{\text{-1}}\text{ dried soil})=\frac{\text{Colony number per plate}\times \text{Dilution fold}\times \text{10}}{\text{1 }-\text{ Soil water content}} \end{array}$$

### Antagonistic activities of bacteria against *F. oxysporum in vitro*

The antagonistic activities of bacterial strains against the pathogenic *F. oxysporum* (isolate no. B3S1) of soybean root rot were tested *in vitro* through dual culture assays on 1/2 Trypticase Soy Agar Medium (TSA, tryptone 15 g, peptone from soybean 5 g, agar 15 g, NaCl 5 g, distilled water 1,000 ml, and pH 7.0). Specifically, tested strains were streaked at both sides of the TSA plate when the fungal plugs with a 5-mm diameter were placed in the center. Plates that were only inoculated with *F. oxysporum* served as negative controls. Each bacteria strain included five plates, and there were three independent replicates. All plates were cultured in the dark at 25°C until the control plates were fully covered with pathogen mycelia. The radial growth of the pathogen was measured with a Vernier caliper. The percent inhibition of radial growth (PI) was calculated according to the equation below:


$$\begin{array}{l} \text{PI}\left(\text{\%}\right)=\frac{\left(\text{R}1\text{ R}2\right)}{\text{R}1}\times \;100 \end{array}$$

where R1 was the radial growth of pathogens in the controls and R2 was the radial growth of *F. oxysporum* in the dual culture with the antagonistic effect. Bacteria strains with antagonistic activity were screened for further assays.

### Treatment of soybean seeds with IRHB3

Seeds of soybean cultivar Nandou12, the moderate susceptible cultivar to the pathogenic *F. oxysproum* of soybean root rot, were sequentially surface-sterilized with 10% H_2_O_2_ (V/V) for 10 min, washed three times with sterile water, and then transferred into a budding box with the size of 120 mm × 50 mm filled with sterilized vermiculite.

**Figure 1:**
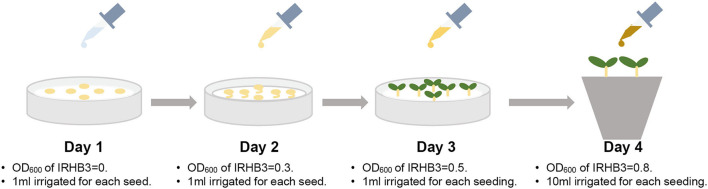
Schematic diagram of inoculation of IRHB3.

For bacterial treatments, the strain IRHB3 was sub-cultured in lysogenic broth (LB, tryptone 10 g, NaCl 10 g, yeast extract 5 g, distilled water 1,000 ml, and pH 7.0) liquid medium at 150 rpm·min^−1^ for 12 h at 28°C. After centrifuging at 5,000 rpm·min^−1^ for 15 min, the bacterial pellet was collected, washed two times with ddH_2_O, and finally resuspended in ddH_2_O. The OD_600_ value of the bacterial suspension was adjusted and then used for watering soybean seeds as treatment. The seeds were sequentially treated with IRHB3 for 3 days as follows in Figure 1: on the first day, seeds were irrigated with sterile water; on the second day, seeds were irrigated with bacterial suspension of IRHB3 with the OD_600_ value of 0.3; on the third day, seeds were continuously treated with bacterial suspension of IRHB3 with the OD_600_ value of 0.5. Each soybean seed was inoculated with 1 ml of bacterial suspension on the corresponding days. On the fourth day, all germinated seeds were transplanted into nutrient soils for pot experiments and each seed was supplemented with 10 ml of the bacterial suspension with an OD_600_ value of 0.8. Seeds were treated with equivalent ddH_2_O instead of IRHB3 for continuous 4 days as controls.

### Control efficiency of IRHB3 on soybean root rot

For pathogen inoculation of soybean root rot, *Fusarium oxysporum* B3S1 was inoculated using sorghum seed inoculation as described by Chang et al. (2020) and Yin et al. (2021) with some improvements. In brief, *F. oxysporum* B3S1 was cultured in potato dextrose agar medium (PDA, potato 200 g, glucosum anhydricum 10 g, agar 15 g, distilled water 1,000 ml, and pH 7.0) for 5 days. Totally 10 mycelial plugs were inoculated into sorghum seeds that had been autoclaved two times before. The infected sorghum seeds were incubated at 25°C in the dark for 7–10 days with gently shaking every day, so that they were adequately colonized. After that, colonized sorghum seeds were dried at 25°C for 6 h in an oven and then ground into colonized seed powder. The diseased soils were prepared by fully mixing the colonized seed powder with the autoclaved Pindstrup substrate, when the mixture of sorghum powder without *F. oxypsorum* and autoclaved nutritious substrate was used as control soils. The inoculum density of *F. oxysporum* B3S1 was calculated based on fungal colony counts by dilution plating on the PDA medium of different diseased soils as described above to ensure that the final concentration of 1 × 10^5^ propagules per gram. Plastic pots were filled with either diseased soil or control soil, respectively.

For pot experiment assays of growth promotion and control efficiency, soybean seeds were pretreated with bacterial suspension and germinated as earlier, and then they were transplanted into either diseased or control soils. Five treatments were set up in the pot experiments as follows: (1) “Control,” seeds without IRHB3 pretreatment and planted in healthy control soil; (2) “IRHB3,” seeds pretreated with IRHB3 and then planted in healthy control soil; (3) “*F.o*,” seeds without IRHB3 treatment and planted into *F.o*-inoculated diseased soil; (4) “IRHB3+*F.o*,” seeds pretreated with IRHB3 and then planted into *F.o*-inoculated diseased soil, which was used to test the protective effect of IRHB3 on healthy plants when suffered to *F. oxysporum* invasion; (5) “*F.o*+IRHB3,” the seeds without IRHB3 pretreatment and planted into *F.o*-inoculated diseased soils for 1 day to make pathogen successfully infect, and then each seedling was irrigated with 10 ml of IRHB3 suspension at an OD_600_ value of 0.8, which aimed to test the therapeutic effect of bacterial strain IRHB3 on diseased plants. All pots were arranged in a randomized complete block design in an artificial climate incubator and incubated at an alternative of 16 h light and 8 h darkness at 28°C. Each treatment contained 10 pots, and each pot planted two soybean seedlings. The experiment was independently conducted three times. Each pot received 20 ml of water two times 1 week. After 2 weeks, the soybean seedlings were removed from the pots, and the roots were washed free of soil. The seedling growth parameters were tested. The disease symptoms of soybean root rot were observed, and the survival rate and disease index were calculated according to disease grade on a 0 to 4 scale (Chang et al., 2018).

### Data analysis

Data analysis was performed in DPS 9.01 (Zhejiang University, Hangzhou, China) and GraphPad Prism8 (GraphPad Software, California, USA). Tests for homogeneity of variance and normal distribution were performed before analysis of variance (ANOVA), and then LSD (L) and Waller–Duncan (W) *post-hoc* tests were applied to determine the significant difference among means of the treatment at the significance level of 0.05 or 0.01. The phylogenetic tree was constructed by MEGA 7.0 software. Graphics were generated using GraphPad Prism8.

## Results

### Diversity and composition of the bacterial community in soybean rhizosphere

**Figure 2:**
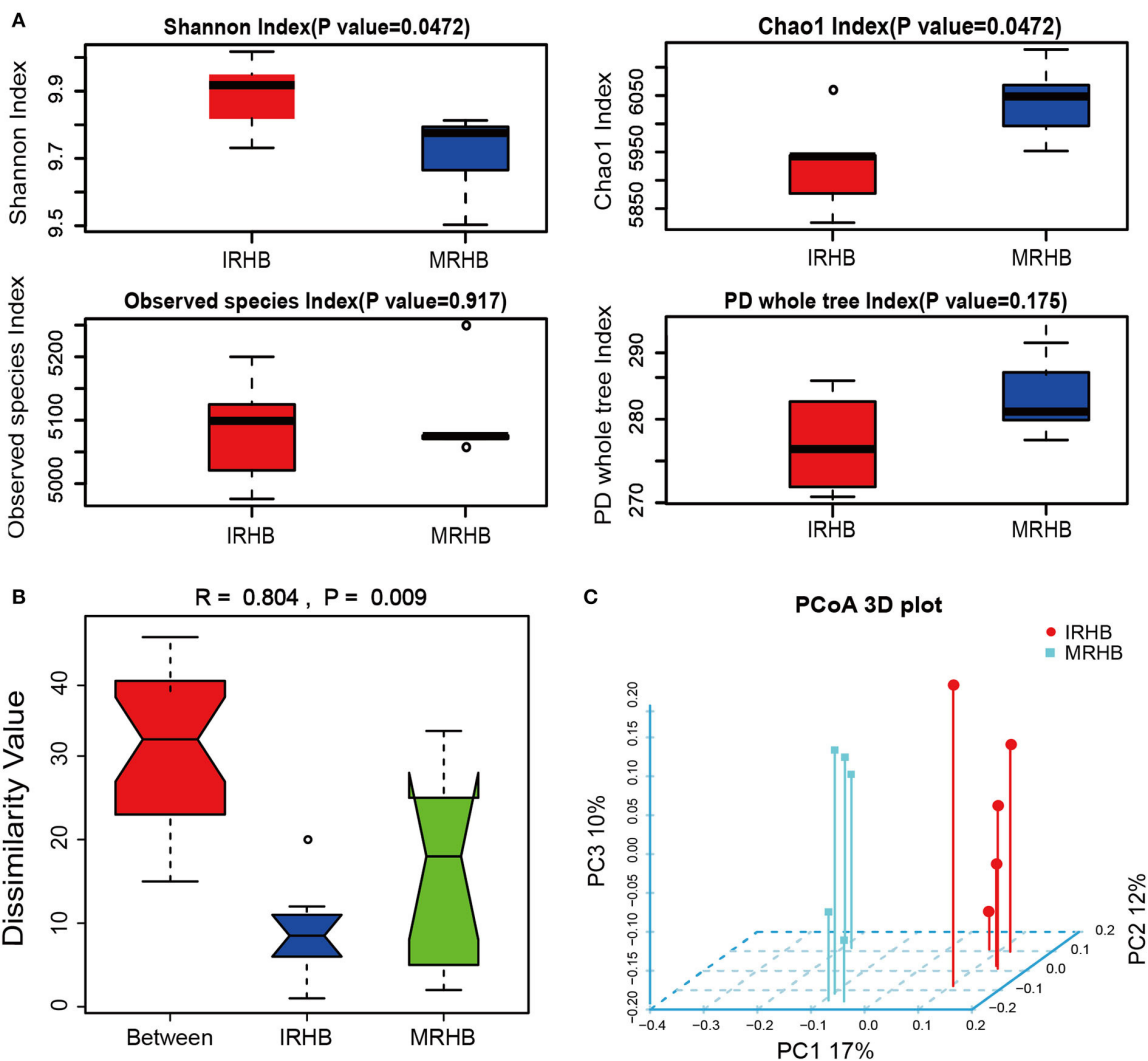
Comparison of the bacterial community structure of soybean rhizosphere soil between intercropping and monoculture based on high-throughput sequencing of *16S rDNA* amplicons. **(A)** Alpha diversity index analysis including Shannon index, Chao1 index, Observed species, and PD whole tree index. **(B)** Anosim similarity analysis, *R* > 0 indicates that the difference between groups is greater than the difference within the group; *P* < 0.05 indicates that the difference between groups is significant. **(C)** Three-dimensional principal coordinate analysis (PCoA) of unweighted-unifrac dissimilarity. IRHB and MRHB stand for the bacterial community of healthy soybean rhizosphere in the maize-soybean relay strip intercropping and soybean monoculture, respectively.

To clarify the differences in the bacterial community diversity of healthy soybean rhizosphere in the maize-soybean relay strip intercropping (IRHB) and soybean monoculture (MRHB), rhizosphere samples of IRHB and MRHB with five biological replicates were subjected to high-throughput *16S rDNA* sequencing (seen in Supplementary Data Sheet S1). As shown in Figure 2, alpha diversity analysis showed that intercropping improved the diversity of the rhizosphere microbial community. Shannon index showed that the diversity of the bacterial community in the IRHB was significantly higher than that in the MRHB, and the distribution was more uniform (*P* = 0.0472). The chao1 index showed the total number of OUT detected in the IRHB was relatively less than that in the MRHB (R = 0.804, *P* = 0.009). The observed species index of both samples was between 5,100 and 5,200 with high species diversity, but it was not significantly different between the two cropping patterns (*P* = 0.917). PD whole tree index based on phylogenetic tree considering evolutionary distance had also almost no difference between IRHB and MRHB (*P* = 0.175). These results indicate that maize-soybean relay strip intercropping remarkably optimizes the diversity of the bacterial community in the soybean rhizosphere but fails to increase the total number of bacteria (Figure 2A).

To further analyze the bacterial composition, we found that the intercropping largely changed the composition and structure of the rhizosphere bacteria community. Anosim similarity indicated that the difference between IRHB and MRHB was greater than that within groups (R = 0.804), and the composition of rhizosphere microbes had a remarkable difference between the two samples (*P* = 0.009) (Figure 2B). Meanwhile, there was no overlap in the spatial distribution of the two samples according to PCoA analysis, implying that there is no similarity in the predominant bacteria between IRHB and MRHB (Figure 2C, Supplementary Data Sheet S1).

**Figure 3:**
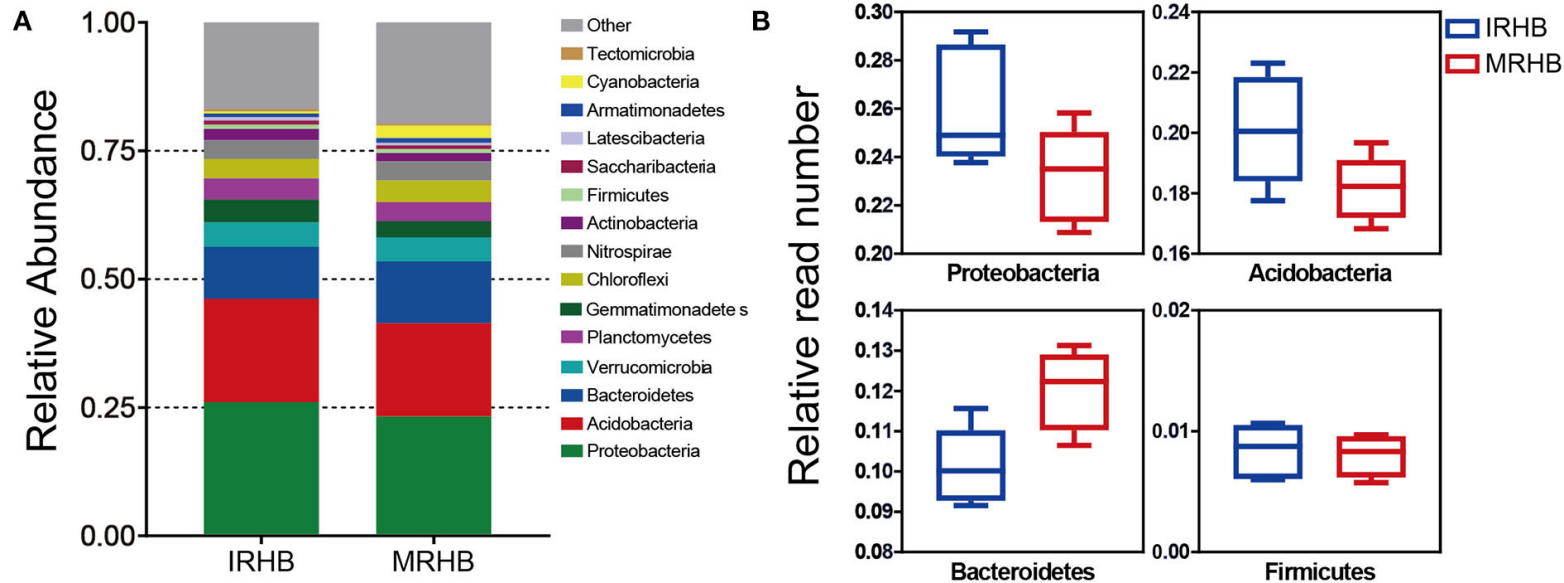
Comparison of relative abundance of rhizosphere bacteria between intercropping and monoculture. **(A)** Relative abundance of the top 15 rhizosphere bacteria in the intercropping (IRHB) and monoculture (MRHB) samples at the phylum level. **(B)** Relative abundance of Proteobacteria, Acidobacteria, Bacteroidetes, and Firmicutes in the intercropping (IRHB) and monoculture (MRHB) samples.

In addition, by comparing the relative abundance of the top 15 rhizosphere bacteria at the phylum level (Supplementary Data Sheet S1), we found that the main contribution of the microbial community was derived from Proteobacteria, Acidobacteria, and Bacteroidetes (Figure 3A). Among them, the relative read numbers of Proteobacteria and Acidobacteria phyla were around 1.2-fold higher in the IRHB than those in MRHB. Moreover, there was also a more abundance of Firmicutes in IRHB rather than in MRHB. However, for the Bacteroidetes phylum, its relative reads in IRHB were reduced by 1.18-fold as compared to those in MRHB (Figure 3B). In short, it is clear that maize-soybean relay strip intercropping could reconstruct the bacterial community of the soybean rhizosphere to increase rhizosphere bacterial diversity.

### Intercropping alters the diversity and structure of culturable bacterial communities of soybean rhizosphere as compared to monoculture

**Figure 4:**
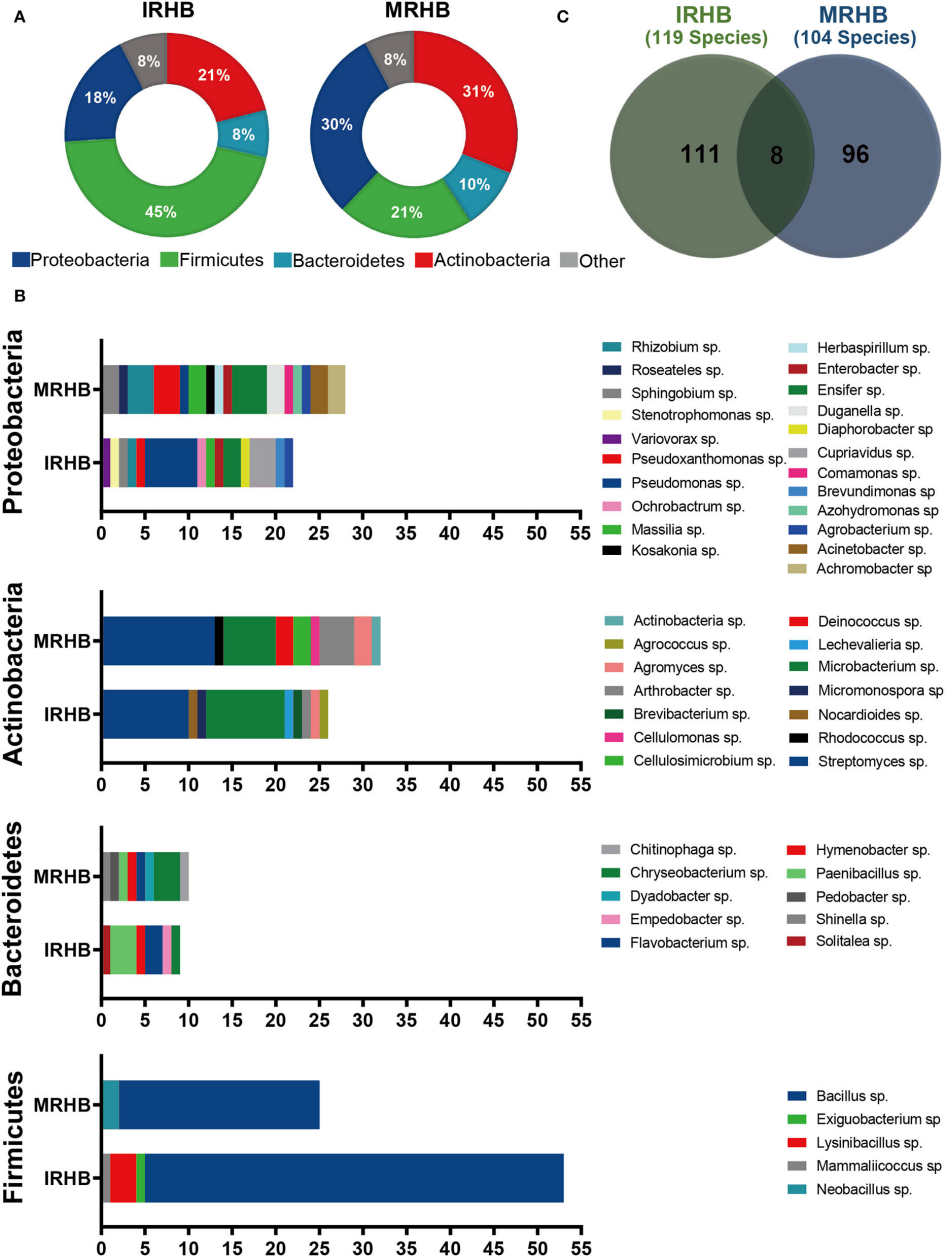
Comparative analysis of culturable bacteria and components in IRHB and MRHB samples. **(A)** The species contribution rate at the phylum level. **(B)** The species structure comparison at the genus level between IRHB and MRHB samples. **(C)** The number of bacteria isolated at the species level.

To further verify the difference in rhizosphere bacterial community between intercropping and monoculture, we isolated and identified the culturable bacteria of soybean rhizosphere from the two cropping patterns and compared the diversity of bacterial community (seen in Supplementary Data Sheet S2). We found that a total of 426 strains of bacteria were isolated, and they were identified as Proteobacteria, Actinobacteria, Bacteroidetes, Firmicutes, and other phyla, and the main four phyla were present in both IRHB and MRHB samples. However, the species contribution rates for each phylum were distinct in both samples. Among them, Firmicutes was the predominant phylum in IRHB with a species contribution rate as high as 45.38% as compared to 25.15% in MRHB (Figure 4A).

At the genus level, 34 and 36 genera were isolated from IRHB and MRHB samples, respectively. For the contribution rate of each genus, *Bacillus* sp. (41.18%), *Streptomyces* sp. (7.56%), *Microbacterium* sp. (6.72%), *Pseudomonas* sp. (5.04%), and *Lysinibacillus* sp. (2.52%) were the main contribution genus for rhizosphere bacterial community in IRHB, while *Bacillus* sp. (19.23%), *Streptomyces* sp. (12.50%), *Microbacterium* sp. (5.77%), *Rhizobium* sp. (4.81%), and *Ensifer* sp. (3.85%) contributed more than other genera in MRHB (Figure 4B).

At the species level, 119 species were isolated from IRHB when compared with 104 species from MRHB. However, it was worth noting that only eight species were shared from both samples (Figure 4C), and they were the least species isolated from Firmicutes in all four phyla. As shown in Figure 4B, it is clearly shown that *Bacillus* sp. genera in the Firmicutes phylum were the most dominant genus in both IRHB and MRHB samples. There were 49 species of *Bacillus* sp. isolated from IRHB with a species contribution rate of 41.18%, whereas 20 species of *Bacillus* sp. from MRHB with a species contribution rate of 19.23%. In addition, *Lysinbacillus* sp. was also the rich and distinct genus of Firmicutes phylum from IRHB rather than MRHB. *Microbacteria* sp. and *Streptomyces* sp., as the second-rich bacteria in the Actinobacteria phylum, were also isolated from both samples. For *Microbacterium* sp., the species contribution to IRHB (8 species) was higher than MRHB (6 species), but for *Streptomyces* sp., the species contribution to IRHB (9 species) was less than MRHB (13 species). In the Bacteroidetes phylum, *Flavobacterium* sp. and *Paenibacillum* sp. as the sharing genera had much higher species contribution in IRHB than those in MRHB. In addition, *Pseudomonas* sp. in the Proteobacteria phylum was a significantly different genus between IRHB and MRHB, and it was much richer in IRHB (6 species) than MRHB (1 species). In general, the culturable bacteria of soybean rhizosphere in both intercropping (IRHB) and monoculture (MRHB) samples were consistent with those based on the high-throughput sequencing analysis, and there was higher diversity of rhizosphere bacteria in intercropped soybean than that in monocultured soybean. And meanwhile, *Bacillus* sp., *Flavobacterium* sp., *Microbacterium* sp., *Paenibacillum* sp., *Pseudomonas* sp., and *Streptomyces* sp. might be preferentially recruited by intercropping or monoculture.

**Figure 5:**
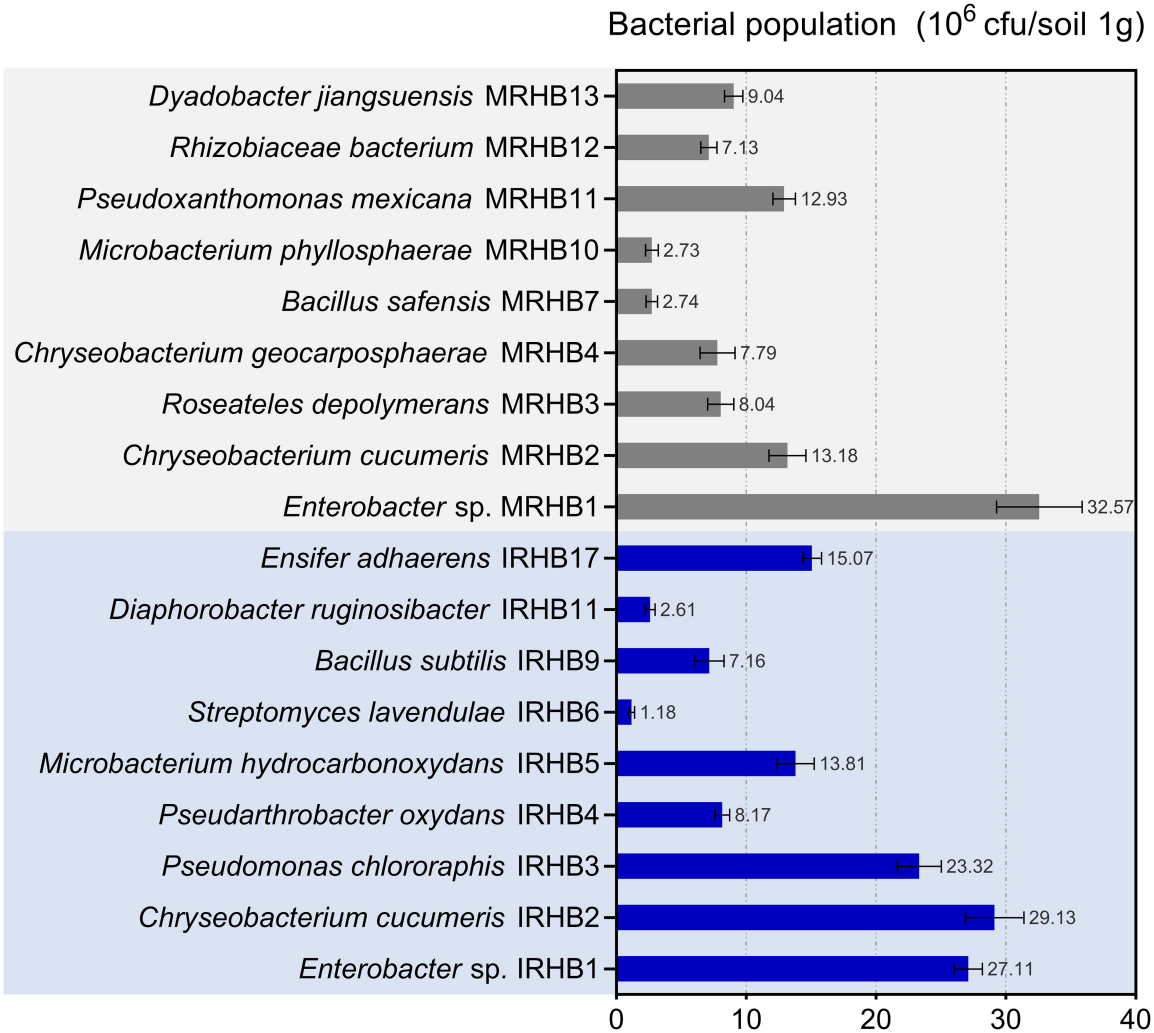
The dominant bacteria species in the rhizosphere soil of IRHB and MRHB samples (Top 9).

To find out the difference in the abundance of rhizosphere bacteria, we also compared the richness of the top nine bacterial species in both samples. As shown in Figure 5, *Enterobacter* sp. was the only shared species of all 9 species and was extremely abundant in both IRHB and MRHB. *Pseudomonas chlororaphis* IRHB3, *Microbacterium hydrocarbonoxydans* IRHB5, *Streptomyces virginiae* IRHB6, and *Bacillus subtilis* IRHB9 were the dominant bacterial population in IRHB with the contents of 23.32 × 10^5^ cuf·g^−1^ soil, 13.18 × 10^5^ cfu·g^−1^ soil, 1.18 × 10^5^ cfu·g^−1^ soil, and 7.16 × 10^5^ cfu·g^−1^ soil, respectively. In contrast, for MRHB, only *Bacillus safensis* MRHB7 and *Microbacterium phyllosphaerae* MRHB10 were listed in the dominant population with the content of 2.73 × 10^5^ cfu·g^−1^ soil and 2.74 × 10^5^ cfu·g^−1^ soil (Figure 5). Thus, since *Pseudomonas* sp., *Streptomyces* sp., *Microbacterium* sp., and *Bacillus* sp. have been well-known as potential biocontrol bacteria, the high abundance and better composition of these bacteria in IRHB might be more stable and resistant when infected by pathogens.

### Antifungal capabilities of soybean rhizosphere bacteria differed from intercropping and monoculture

**Figure 6:**
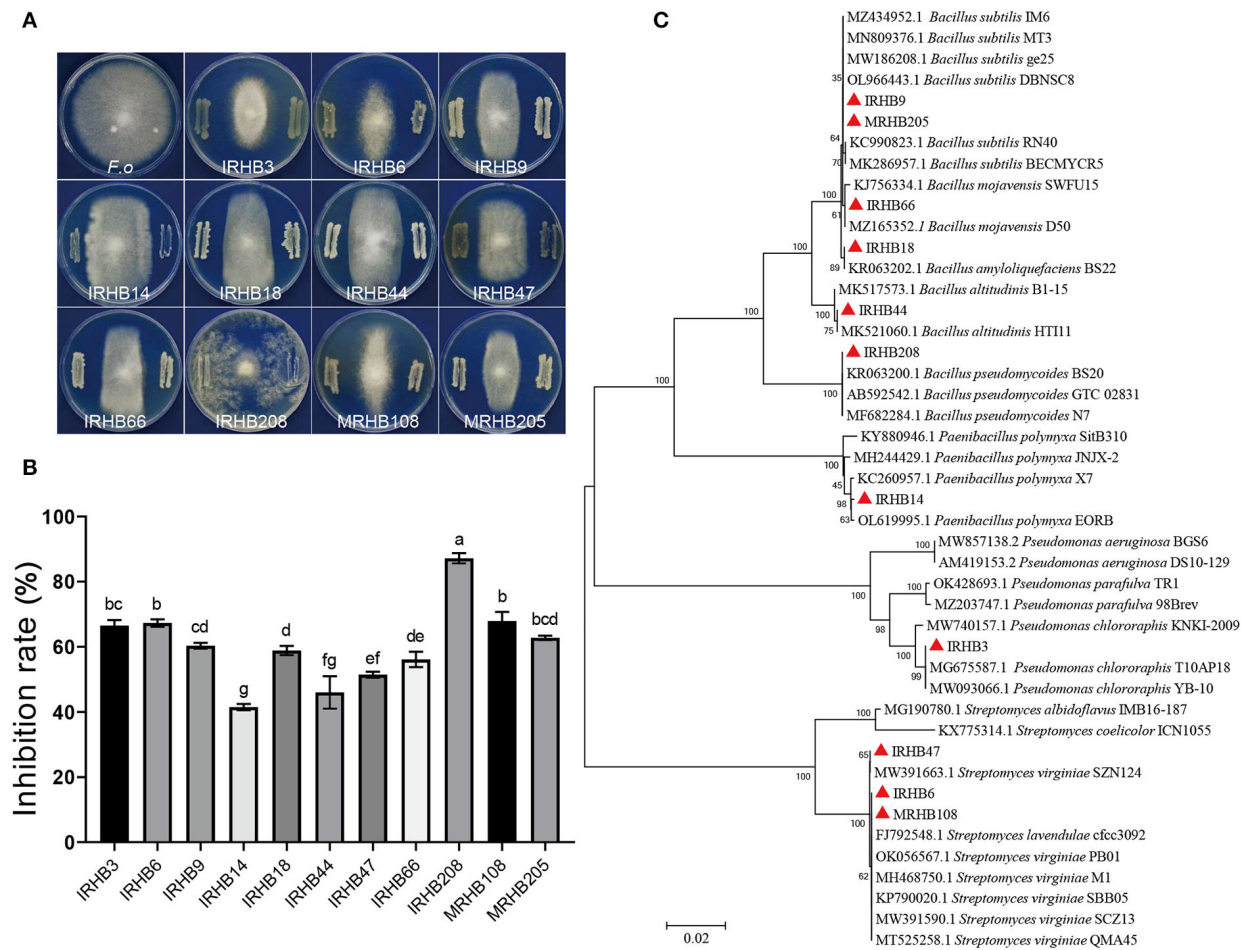
Antagonistic bacterial strains screened by soil-borne pathogens *F. oxysporum* in IRHB and MRHB samples. **(A)** Dual culture assays of *F.oxysporum* verus antagonistic bacteria *in vitro*. **(B)** Inhibition rate of antagonistic bacteria to *F.oxysporum* mycelial growth. **(C)** Phylogenetic tree of 11 antagonistic bacteria based on *16S rDNA* sequences. *F.o* means the pathogen *F. oxysporum* of soybean root rot. Data represent mean ± SEM. Different lowercase letters indicate significant differences (*P* < 0.05).

To confirm the biocontrol potential of isolated rhizosphere bacteria, the dual culture assays were conducted using 223 bacterial strains versus *Fusarium oxysporum* B3S1 (*F.o*) *in vitro* (seen in Supplementary Data Sheet S3). We found that 11 species exhibited antagonistic activities against *F. oxysporum* at different levels (Figure 6A), and among them, 9 strains including *Pseudomonas chlororaphis* IRHB3, *Streptomyces lavendulae* IRHB6, *Bacillus subtilis* IRHB9, *Paenibacillus polymyxa* IRHB14, *Bacillus amyloliquefaciens* IRHB18, *Bacillus altitudinis* IRHB44, *Streptomyces virginiae* IRHB47, *Bacillus mojavensis* IRHB66, and *Bacillus pseudomycoides* IRHB208 were isolated from IRHB sample (Figure 6C). Their inhibition rate on the mycelial growth of *F. oxysporum* ranged from 41.49 to 87.18% (Figure 6B). In contrast, in the MRHB sample, only *Streptomyces virginiae* (MRHB108) and *Bacillus subtilis* (MRHB205) (Figure 6C) displayed good inhibition effects on *F. oxysporum* with inhibition rates of 67.87 and 62.75%, respectively (Figure 6B). Thus, considering the diversity and abundance of antagonistic bacteria, intercropped soybean might tend to recruit a variety of beneficial bacteria and particularly increase their abundance when compared with soybean monoculture. Interestingly, the strain IRHB3, identified as *Pseudomonas chlororaphis*, was the only *Pseudomonas* species among 11 antagonistic bacteria and was specially isolated in the IRHB sample. The mycelial growth inhibition rate of IRHB3 to *F. oxysporum* was 66.50%, displaying strong antibacterial ability. Moreover, Since IRHB3 was one of the most dominant bacteria in IRHB with the abundance of 2.23×10^6^ cfu·g^−1^ (Figure 5), we therefore have a strong interest in the role of IRHB3 in the maize-soybean relay strip intercropping.

### IRHB3 improved soybean resistance against *F. oxysporum*

**Figure 7:**
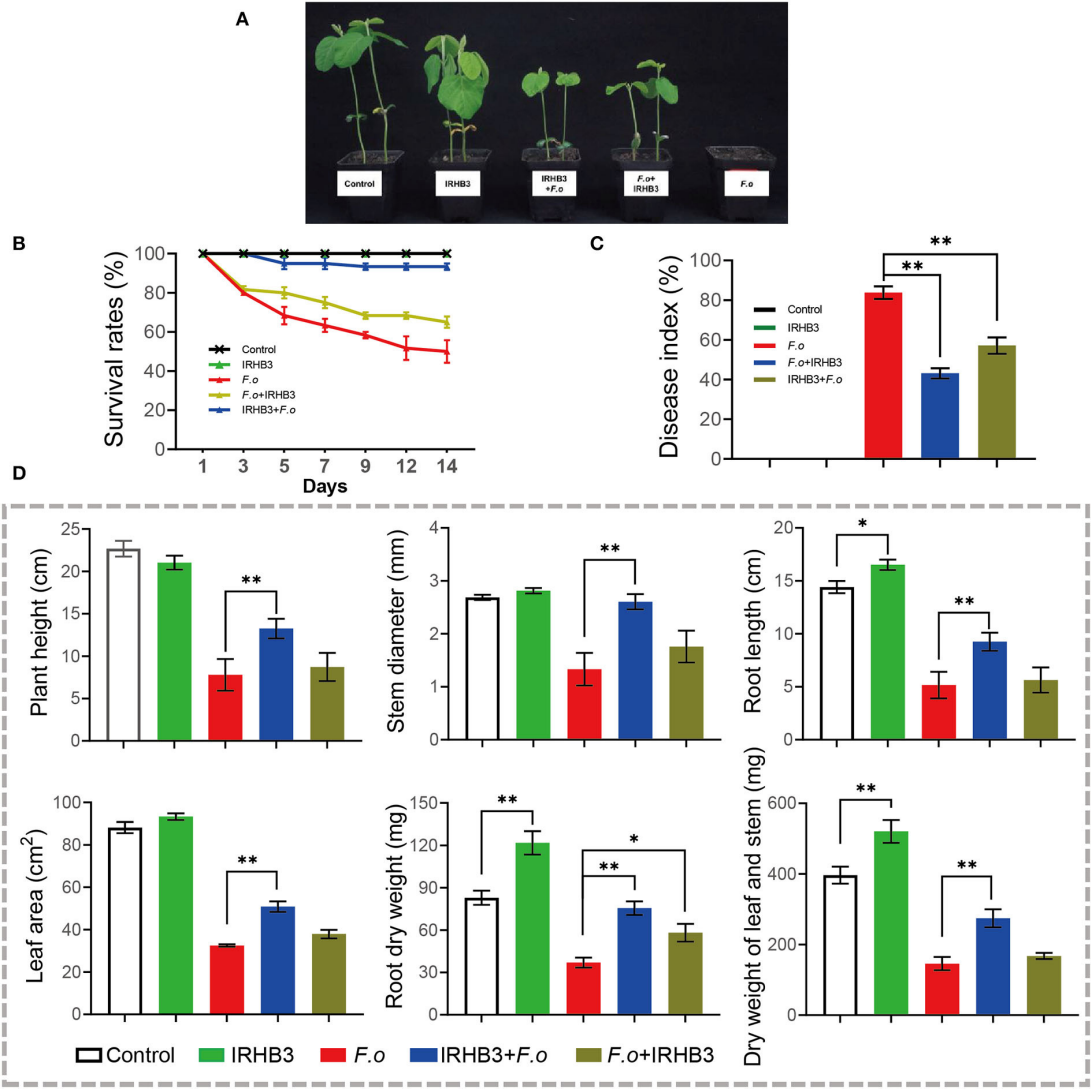
Effects of IRHB3 treatments on soybean growth and the occurrence of soybean root rot. **(A)** Soybean seedlings at 14 days post-treatment. **(B)** The survival rate of soybean seedlings. **(C)** Disease index of soybean root rot. **(D)** Growth parameters of soybean seedlings include plant height, stem diameter, root length, leaf area, dry weight of stem and leaf, and root dry weight. Data represent mean ± SEM. Asterisks indicate significant differences (*P < 0.05, **P < 0.01). The experiments were conducted three times and showed similar results.

We assessed the control effects of IRHB3 on soybean root rot, mainly caused by *F. oxysporum*. As shown in Figure 7A, compared with single *F. oxysporum* inoculation, IRHB3 application, no matter before or after *F. oxysporum* inoculation, largely alleviated the disease symptoms of soybean seedlings. To compare the survival rate with different treatments, IRHB3 had almost no influence on the survival of soybean rather than control, but it significantly improved the survival of soybean seedlings that suffered from *F. oxysproum* infection. Seed pretreated with IRHB3 significantly reduced *F. oxysporum* infection and increased the survival rate by 30% (Figure 7B). Furthermore, the disease index of soybean root rot was also affected by IRHB3 to a large extent (Figure 7C). *F. oxysporum* infection caused the disease index as high as 85%, whereas IRHB3 application before or after pathogen infection largely decreased the disease index by 20–40%, which was much more effective for the pretreatment with IRHB3 (Figure 7C).

In addition, we also found that seeds pretreated with IRHB3 improved seedling growth that was severely inhibited by *F. oxysproum* invasion (Figure 7D). After 14 days of pathogen inoculation, IRHB3 dramatically increased the root length by 16%, dry weight of leaf and stem by 31%, and root dry weight by 48% when compared with controls. Meanwhile, IRHB3 application before pathogen inoculation greatly improved plant height, root length, stem diameter, leaf area, dry weight of aboveground tissues, and root dry weight, whereas IRHB3 supplement after pathogen inoculation merely increased the root dry weight (Figure 7D). These results indicate that IRHB3 might play a more important role in preventing pathogen invasion but not inhibiting its spread into the soybean tissues.

## Discussion

The rotation or intercropping of cereal grains with legumes has widely been accepted as a sustainable agricultural strategy, and it plays an important role in improving soil quality and regulating the rhizosphere microbial community. In the rhizosphere, the diversity and structure of the microbial community can affect soil-borne disease and plant health to a large extent. In particular, rhizosphere-beneficial microbes often inhibit or directly kill pathogens in diverse ways thus leading to the reduction of soil-borne diseases. Our previous study demonstrated that maize-soybean relay strip intercropping clearly suppressed soybean root rot and altered the diversity and composition of the pathogenic *Fusarium* species rather than soybean monoculture. Furthermore, this intercropping also changed the structure and abundance of cultural *Fusarium* and the biocontrol *Trichoderma* species of soybean rhizosphere (Chang et al., 2020). Since bacteria are the key composition and regulator of the rhizosphere microbial community, it is necessary to investigate the difference in the bacterial community of soybean rhizosphere between intercropping and monoculture, and particularly explore the beneficial bacteria that interacted with the soil-borne pathogen.

Plants can provide rich carbon and energy to soil biota through root exudates, and create a unique micro-environment for bacteria to colonize in the rhizosphere soil around their roots (Bais et al., 2006). In addition, plants can also recruit the microbiome from other plants around them (Gross, 2022). From this point, the complicated and rich plant diversity is beneficial to constructing a more stable and healthy soil microbial community (Eisenhauer et al., 2010). Intercropping, which is characterized by planting two crops in the same fields, increases the plant biodiversity in the agricultural system, and it also has big potential on affecting soil microbial diversity and structure as compared to crop monoculture. For example, sugarcane and soybean intercropping changes the soil fungal community structure and increases the abundance of the main fungus *Trichoderma* (Lian et al., 2018); mulberry and alfalfa intercropping significantly increases microbial metabolic activity, richness index, and dominance index, and changes the carbon source types of soil microbial utilization (Zhang et al., 2015). Many studies have proved that due to the complex interaction within the microbial community, a high level of microbial diversity is often more conducive to resisting pathogen attack (Van Elsas et al., 2012). Wei et al. (2015) reported that the diversity of resident communities directly affected the spread of bacterial wilt disease in the tomato rhizosphere. In this study, the integrated results of high-throughput sequencing and the isolation of culturable bacteria demonstrated that maize-soybean relay strip intercropping significantly changed the bacterial community structure of soybean rhizosphere and increased the diversity of rhizosphere bacteria, and especially increased the abundance of beneficial bacteria Actinobacteria and Proteobacteria at phylum levels. It has been reported that Rhizosphere Actinobacteria, Proteobacteria, and Firmicutes are the key phylum of bacteria that affect crop health and contribute to disease suppression (Mendes et al., 2011; Carlstrom et al., 2019). The abundance change of Firmicutes and Actinobacteria in the tomato rhizosphere aggravates bacterial wilt disease (Lee et al., 2021). In addition, disease-resistant varieties often tend to recruit more Actinobacteria, Proteobacteria, and Firmicutes in their rhizosphere (Lazcano et al., 2021). Taken together, our results provide further evidence that intercropping can change the structure of the soil microbial community (Boudreau, 2013; Lian et al., 2018; Lv et al., 2018).

Moreover, the rich plant diversity not only has beneficial effects on the richness and activity of soil microorganisms but also enhances plant resistance to pathogens by recruiting beneficial bacterial communities (Mendes et al., 2011; Latz et al., 2012). When these beneficial microorganisms successfully colonize the plant rhizosphere, on the one hand, they can directly promote plant growth through bio-fertilization (Song et al., 2021, 2022), stimulation of root growth (Glick, 1995; Glick et al., 1995; Dhar Purkayastha et al., 2018), rhizo-remediation (Lugtenberg and Kamilova, 2009), and plant stress control (Trivedi et al., 2020), and on the other hand, rhizosphere microbes can also indirectly protect plant health by antibiosis (Gu et al., 2020; Shalev et al., 2022), induction of systemic resistance (Pieterse et al., 2014; Lee et al., 2021), and competition for nutrients and niches (Barber and Elde, 2015; Humphrey et al., 2020; Shalev et al., 2022). A lot of evidence show that *Pseudomonas* sp., *Streptomyces* sp., *Bacillus* sp., *Microbacillus* sp., and *Flavobacterium* sp. as common rhizosphere growth-promoting bacteria (PGPR) can promote plant growth and inhibit the occurrence of soil-borne disease (Kwak et al., 2018; Abbasi et al., 2021). Especially *Pseudomonas* sp., *Streptomyces* sp., and *Bacillus* sp., which were often used as the key research objects of biological control, are widely used as biological repair agents in agricultural production (Antoun and Prévost, 2005; Pieterse et al., 2014; Qin et al., 2017). Since beneficial bacteria could promote crop growth and affect health (Berendsen et al., 2012; Trivedi et al., 2020), as a countermeasure, plants usually actively recruit beneficial soil microorganisms in their rhizosphere to resist the attack of pathogens (Liu et al., 2021; Yin et al., 2021). In our study, we found 72 species of potential PGPR in the rhizosphere of intercropped soybean and 40 species in monoculture. Intercropping significantly increased the diversity of beneficial bacteria in the soybean rhizosphere, especially raising the diversity of *Bacillus* and *Pseudomonas*. Moreover, the population of PGPR recruited by intercropping was nine-fold higher than that by monoculture. Hence, the alliance of beneficial bacteria in the intercropping with higher diversity and more abundance forms powerful microbial barriers in the soybean rhizosphere to protect plant health and overcome pathogen invasion.

Researches demonstrate that most PGPR can restrict the growth and development of pathogens by the production of a variety of antibacterial compounds (Gu et al., 2020). These compounds lead to cell lysis, potassium ion leakage, destruction of membrane structural integrity, inhibition of mycelium growth, inhibition of spore germination, and protein biosynthesis (Hyder et al., 2020). For example, *Bacillus* strain BPR7 can produce IAA, siderophore, phytase, organic acid, ACC deaminase, cyanogens, lytic enzymes, oxalate oxidase, and so on, and these substances strongly inhibit the growth of many pathogens, such as *F. oxysporum, F. solani, Sclerotinia sclerotiorum, Macrophomina phaseolina, Rhizoctonia solani*, as well as *Colletotricum* sp. (Kumar et al., 2012). In our study, the antagonistic assay *in vitro* showed that 11 out of 223 isolated bacteria strains had good inhibition effects on the growth of *F. oxysporum*, and they were mainly identified as *Pseudomonas* sp., S*treptomyces* sp., and *Bacillus* sp., which have been fully demonstrated to inhibit pathogens (Weller, 2002; Van Elsas and Costa, 2007; Yin et al., 2013). However, among these antagonistic bacteria, nine strains were from intercropping and two strains from monoculture, indicating more diverse and synthetic beneficial microbial communities in the intercropped soybean rhizosphere. These diverse and synthetic antagonistic microbes might provide much stronger disease resistance and growth promotion to host plants than single species (Finkel et al., 2017; Kehe et al., 2019). This hypothesis is also supported by Lee et al. (2021) that proved a synthetic community of four strains activated a stronger immune response against *R. solanacearum*. Furthermore, not only for more species but also the population of antagonistic bacteria gathered in the rhizosphere of intercropping soybean was more abundant with 4.547 × 10^6^ cfu·g^−1^ in this study. It is reported that the content of beneficial microorganisms greater than or equal to 10^5^ cfu·g^−1^ could effectively act on plants (Pieterse et al., 2014; Liu et al., 2021). Although two strains *Streptomyces virginiae* (MRHB108) and *Bacillus subtilis* (MRHB205) in soybean monoculture had also antagonistic abilities, the population could not provide effective and multifunctional service for host plants. Therefore, we predict that maize-soybean relay strip intercropping is capable of deploying multiple antagonistic bacteria to confer much higher resistance of soybean to soil-borne diseases than monoculture with limited strains.

Some species of *Pseudomonas* play an important role in plant health and resistance to soil-borne diseases. Previous studies have shown that in the interaction between *Pseudomonas* and host plants, *Pseudomonas* can produce a large number of antibiotics to enhance rhizosphere competitiveness (Poritsanos et al., 2006; Gross and Loper, 2009), and successfully colonized *Pseudomonas* can promote the increase of bacterial community by forming biofilm (Danhorn and Fuqua, 2007). In addition, some *Pseudomonas* species are also proved to induce plant systemic immunity through root colonization in *Arabidopsis thaliana* (Pieterse et al., 2014), tobacco (Zboralski and Filion, 2020), and other plants to resist the infection of a variety of pathogens. In this study, we also screened one strain of *Pseudomonas* and *Pseudomonas chlororaphsi* IRHB3, which was the most dominant bacteria from intercropped soybean rhizosphere with the strongest antagonistic effect against *F. oxysporum*. We found that IRHB3 not only largely prevented *F. oxysporum* infection causing soybean root rot but also alleviated the disease symptoms, implying its dual function of protection and therapeutics in disease control. And meanwhile, compared with controls, IRHB3 application caused soybean seedlings much shorter and stronger, especially in the growth and development of soybean roots. This indicates that IRHB3 might be one kind of typical rhizosphere growth-promoting bacteria (Zamioudis et al., 2013). Zamioudis et al. (2013) reported that *Pseudomonas* Simiae WCS417 derived auxin-dependent root structural changes, promoted plant growth, and induced lateral root production and root hair formation. However, how IRHB3 colonizes in the rhizosphere or endosphere of soybean and how it regulates soybean growth and resistance need to be elucidated in future. Anyway, rhizosphere-beneficial bacteria recruited by intercropped soybean make a great contribution to resisting soil-borne diseases. This is also supported by our data on the disease incidence of soybean root rot at the full bloom (R2) stage of soybean when this disease frequently occurred which was also lower in the intercropping than in soybean monoculture. Thus, our research reveals the reason why maize-soybean relay strip intercropping can effectively inhibit soil-borne diseases, and also provides new evidence for crop intercropping to regulate microbial communities to promote plant health.

In conclusion, we demonstrated that maize-soybean relay strip intercropping increased the diversity of the rhizosphere soil bacterial community and recruited a large number of beneficial bacteria such as *Pseudomonas* sp., *Bacillus* sp., and *Streptomyces* sp., and *Microbacterium* sp. to settle in the soybean rhizosphere. These bacteria help soybean seedlings not only resist soil-borne diseases by antagonizing pathogens, mainly *F. oxysporum*, but also promote plant growth. In particular, *Pseudomonas chlororaphsi* IRHB3 was identified as the most dominant species from intercropped soybean rhizosphere and displayed the strongest antagonism against *F. oxysporum*. This potential biocontrol bacterial strain not only had preventive and therapeutic effects on *Fusarium* root rot but also significantly promoted the root growth and seedling development of soybean. However, how these beneficial bacteria are selected by the host, their colonization pathway, and the mechanism by which they act on the host are still unclear, and will be further uncovered in the following study.

## Data availability statement

The data presented in the study are deposited in the NCBI database, accession numbers: OP364493–OP364720.

## Author contributions

XC supervised projection and wrote and edited the manuscript. DW, YH, YZ, and XZ conducted experiments, data analysis, and wrote the original draft. XW and CS reviewed and edited the manuscript. GG and MZ conceived and designed the research. HC, TL, and CY analyzed data and characterized. WC and WY supervised and acquired funding. All authors have reviewed and approved the submission of our manuscript.

## Funding

This work was supported by the Natural Science Foundation of Sichuan Province (2022NSFSC0173), the National Natural Science Foundation of China (31801685), and the Agricultural Science and Technology Innovation Program (CAAS-ASTIP).

## Conflict of interest

The authors declare that the research was conducted in the absence of any commercial or financial relationships that could be construed as a potential conflict of interest.

## Publisher's note

All claims expressed in this article are solely those of the authors and do not necessarily represent those of their affiliated organizations, or those of the publisher, the editors and the reviewers. Any product that may be evaluated in this article, or claim that may be made by its manufacturer, is not guaranteed or endorsed by the publisher.

## Supplementary material

Supplementary Figure S1 Growth parameters of soybean and disease incidence of root rot between intercropping (IRHB) and monoculture (MRHB) in the fields.

Supplementary Data Sheet S1 Data of PCoA analysis and relative abundance (top 15 phyla) of soybean rhizosphere bacterial communities from intercropping and monoculture through high-throughput sequencing.

Supplementary Data Sheet S2 Identification and species contribution of cultural rhizosphere bacteria from intercropped and monocultured soybean.

Supplementary Data Sheet S3 Screening, identification, and activities of rhizosphere antagonistic bacteria against *F. oxysporum*.
