# Supplementary material for: Maize-soybean relay strip intercropping reshapes the rhizosphere bacterial community and recruits beneficial bacteria to suppress Fusarium root rot of soybean
Source: Front Microbiol. 2022 Oct 26;13:1009689. doi: 10.3389/fmicb.2022.1009689 (PMC9643879; doi:10.3389/fmicb.2022.1009689)
Supplement: Supplementary Figure S1 — Growth parameters of soybean and disease incidence of root rot between intercropping (IRHB) and monoculture (MRHB) in the fields. [file Image_1.pdf]

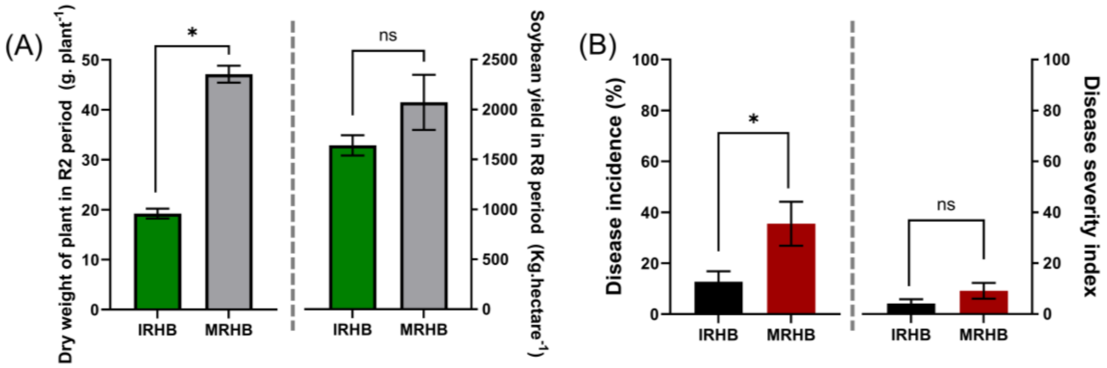

Figure S1 Growth parameters of soybean and disease incidence of root rot between intercropping (IRHB) and monoculture (MRHB) in the fields.

(A) Biomass of soybean at the full bloom (R2) period (left) and the final yield at the full maturity (R8) period (right) ; (B) Disease incidence (left) and disease index (right) of soybean root rot.
